# The Secretome of Alginate-Encapsulated Limbal Epithelial Stem Cells Modulates Corneal Epithelial Cell Proliferation

**DOI:** 10.1371/journal.pone.0070860

**Published:** 2013-07-24

**Authors:** Bernice Wright, Andrew Hopkinson, Martin Leyland, Che J. Connon

**Affiliations:** 1 University of Reading, School of Pharmacy, Reading, Berkshire, United Kingdom; 2 University of Nottingham, Division of Ophthalmology and Visual Sciences, Queen's Medical Centre Campus, Nottingham, United Kingdom; 3 Royal Berkshire Hospital, Reading, Berkshire, United Kingdom; Instituto Butantan, Brazil

## Abstract

Limbal epithelial stem cells may ameliorate limbal stem cell deficiency through secretion of therapeutic proteins, delivered to the cornea in a controlled manner using hydrogels. In the present study the secretome of alginate-encapsulated limbal epithelial stem cells is investigated. Conditioned medium was generated from limbal epithelial stem cells encapsulated in 1.2% (w/v) calcium alginate gels. Conditioned medium proteins separated by 1-D gel electrophoresis were visualized by silver staining. Proteins of interest including secreted protein acidic and rich in cysteine, profilin-1, and galectin-1 were identified by immunoblotting. The effect of conditioned medium (from alginate-encapsulated limbal epithelial stem cells) on corneal epithelial cell proliferation was quantified and shown to significantly inhibit (*P*≤0.05) their growth. As secreted protein acidic and rich in cysteine was previously reported to attenuate proliferation of epithelial cells, this protein may be responsible, at least in part, for inhibition of corneal epithelial cell proliferation. We conclude that limbal epithelial stem cells encapsulated in alginate gels may regulate corneal epithelialisation through secretion of inhibitory proteins.

## Introduction

Damage to the limbal region of the cornea can reduce or destroy the resident population of limbal epithelial stem cells (LESC) responsible for replenishing the corneal epithelium, consequently leading to limbal stem cell deficiency (LSCD) [1]–[2], and persistent epithelial defects resulting in visual loss and blindness. An established therapy for LSCD involves the transplantation of LESC expanded *ex vivo* on a human amniotic membrane (AM) carrier [2]–[7]. The outcomes from this therapy are, however, variable and due to lengthy pre-clinical manufacturing procedures, requiring specialised GMP facilities, it is economically costly to generate therapeutic LESC. Therefore, there is a need to refine current therapy for LSCD to enhance clinical predictability, practicability and economic viability. Understanding the mechanisms by which LESC repair the damaged cornea is possible using hydrogel matrices as cell-encapsulation devices. Furthermore, these constructs may allow significant improvements in the treatment of LSCD by reducing the length of pre-clinical procedures and allowing controlled release of factors secreted from immobilized LESC to the injured cornea. The premise of the present study is that alginate-encapsulated LESC can secrete growth factor-like peptides which migrate through the gel micro-architecture to mediate regeneration of the damaged corneal surface.

**Figure 1:**
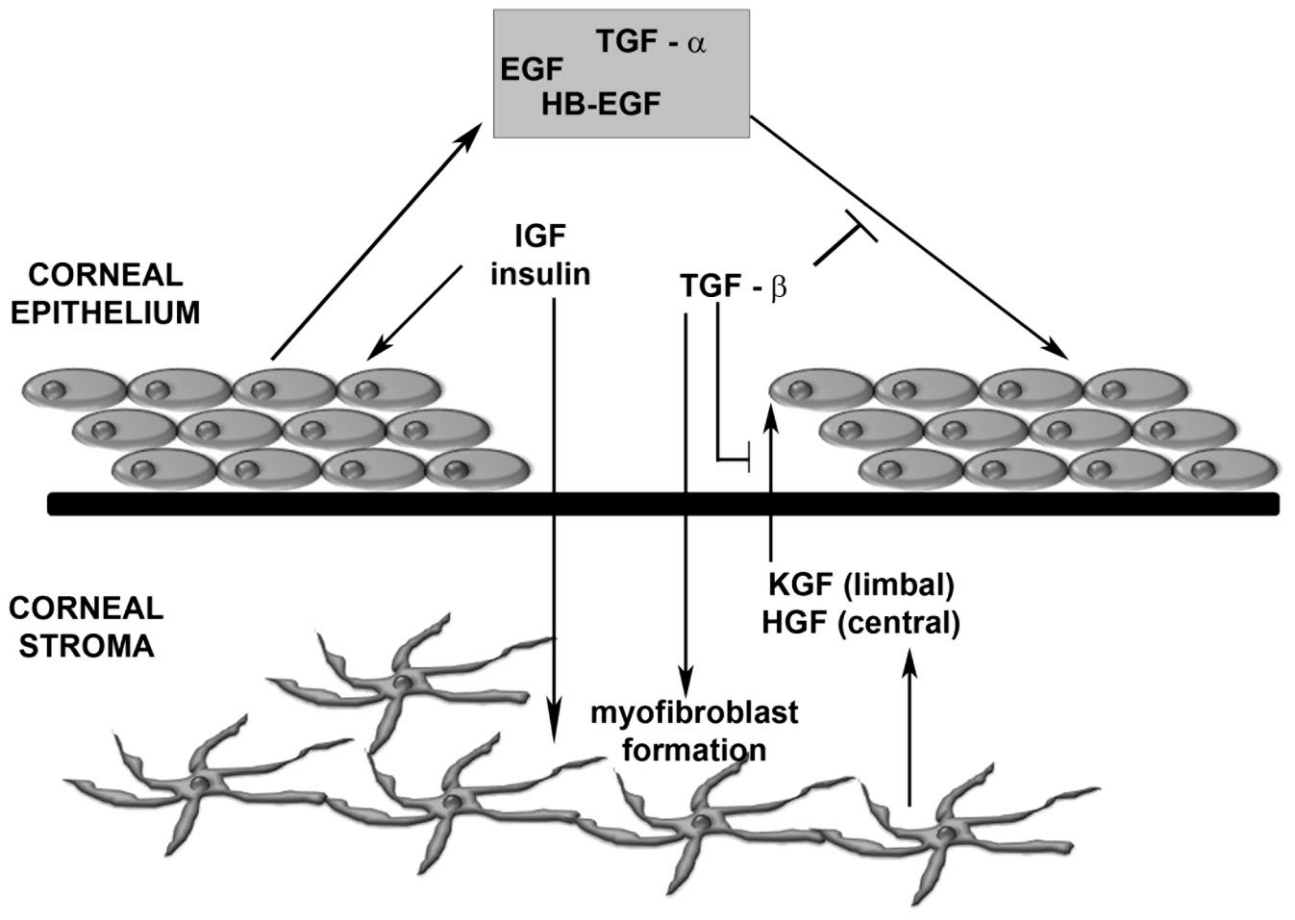
Corneal wound healing. A number of growth factors and cytokines are released following epithelial injury in the cornea. These factors mediate the epithelial-stromal interactions that are critical for wound healing. Keratinocyte growth factor (KGF) and hepatocytes growth factor (HGF) are produced by stromal keratocytes, and interleukin-1 (IL-1) and platelet-derive growth factor (PDGF) are secreted by epithelial cells to regulate stromal responses to injury. Epidermal growth factor (EGF), IGF and transforming growth factor β (TGF-β) play roles in both epithelium and stromal repair. Cross-talk between these factors ultimately determines the outcome of corneal wound healing.

There is little available evidence that cell-cell interactions of transplanted cells drive the healing process. Daya *et al.* (2005) [8] indicated that transplanted LESC may not be incorporated into the exposed, de-epithelialised corneal stromal bed after transplantation. Instead, an established body of evidence demonstrates that corneal wound healing is heavily dependent on growth factors (e.g. epidermal growth factor, keratinocyte growth factor, hepatocyte growth factor) produced by resident differentiated epithelial cells and stromal keratocytes [9]-[11] (**Figure 1**). Therefore proteins secreted by LESC may be responsible for facilitating epithelial repair. This suggests that studying the secretome of LESC may hold the key to elucidating the mechanism of corneal reparation.

The therapeutic importance of the stem cell secretome has recently been highlighted in a number of studies. Specifically, factors released from progenitor cells were reported to beneficially modulate degenerative conditions. For example, mesenchymal stem cell (MSC)-derived molecules were demonstrated to mediate angiogenesis [12]–[13]. Furthermore, trophic and immunomodulatory cytokines secreted from MSC were reported to reverse hepatic failure [14] and were shown to be a potentially effective treatment for ischaemic heart disease [15], as well as regeneration of the central nervous system [16]. Trophic effects of adipose stem cell (ASC) secreted factors (e.g. granulocyte and macrophage colony stimulating factors, interleukins, adipokines) are also protective, and they induce cell differentiation and immunomodulatory effects on a range of endogenous cells/tissues [17]. The ASC secretome is known to impact on the immune and central nervous system, vascularization and cardiac regeneration [17]. Paracrine factors secreted from ASC were shown to lead to distinct effects on the metabolic viability and neuronal cell densities in primary cultures of hippocampal neurons [18]. Effects of the embryonic stem cell (ESC) secretome are less well-characterised than those of MSC and ASC, but profiles of proteins secreted during differentiation of murine ESC were previously reported to be distinct during cardiomyogenesis and neurogenesis [19].

Considering this evidence, in the present study we demonstrate that the conditioned medium generated from primary human LESC immobilized in alginate gels significantly attenuated the proliferation of corneal epithelial cells. We show distinct protein banding patterns between alginate-encapsulated and isolated LESC that highlight differences between proteins at approximately 35, 38, 43 and 55 kDa. We identified secreted proteins that have been reported to both drive (profilin-1 - 12 kDa and galectin-1 – 12–15 kDa) and inhibit (sPARC – 43 kDa) proliferation of epithelial cells. To our knowledge, this is the first study to examine the secretome of LESC.

This research indicates that a pharmacological approach to the treatment of LSCD may be devised by employing the factors that they secrete rather than the cells per se, and that this may eliminate the problems with variability encountered with current therapy for this condition.

## Materials and Methods

### Ethics Statement

Corneoscleral rims were obtained from human cadavers from the Royal Berkshire Hospital following corneal transplants, and used for experiments with approval from the University of Reading Research Ethics Committee. Informed, written consent from the donor or the next of kin was obtained for use of these samples in research.

### Materials

Antibiotics (penicillin/streptomycin, amphotericin B), Whatman chromatography paper, Trypan Blue, Tris-base, acrylamide, acutase, acetic acid, polyvinylidine difluoride (PVDF) membrane and methanol were purchased from Thermo Scientific HyClone (Fisher Scientific: Leicestershire, UK). CnT20 medium was obtained from CellnTec (Advanced Cell Systems: Buckingham, UK). Sodium alginate (viscosity: 15-20 cP, 1% in H_2_O (lit.)), calcium chloride, sodium citrate and sodium chloride were from (Sigma-Aldrich: Poole, UK). Tween®-20, sodium dodecyl sulphate (SDS), 2-mercaptoethanol, ammonium persulphate (AMPS), N,N,N’,N’-tetramethylethylenediamine (TEMED), ethylenediaminetetraacetic acid (EDTA), 2-[2-(4-nonylphenoxy)ethoxy]ethanol (igepal - nonidet P40 (NP40)), phenylmethanesulphonyl fluoride (PMSF), aprotinin, leupeptin, sodium orthovanadate, pepstatin A, silver nitrate, sodium carbonate, sodium acetate, and sodium thiosulphate were also purchased from Sigma. The immortalized human corneal epithelial (HCE) cell-line was from RIKEN BioResource Center (Tsukuba, Ibaraki, Japan) and the human corneoscleral rims were kindly donated by the Royal Berkshire Hospital (Dr Martin Leyland) following cornea transplantations. The enhanced chemiluminescence (ECL) detection system was obtained from Pierce (Thermo Fisher Scientific; Rockford, IL USA). The 14-3-3ζ antibody was from Santa Cruz Biotechnology (Autogen Bioclear Ltd.: Calne, Wilts, UK), the p63 antibody was purchased from Millipore (Dundee, Scotland). sPARC and profilin-1 antibodies were from New England Biolabs (Cell Signaling technology: Hertfordshire, UK). Horseradish peroxidase (HRP)-conjugated anti-rabbit and anti-goat secondary antibodies, galectin-1 antibodies were from R&D systems (Abingdon, UK) and bovine serum albumin was obtained from First Link (Birmingham, UK).

### Methods

#### Isolation of human limbal epithelial cells

LESC were isolated from human corneoscleral rims. Briefly, explants were cultured in CnT20 medium, LESC outgrowths from explants were detached from tissue culture plants using acutase, and these cells were counted before encapsulation in alginate gels.

#### Production of Conditioned Medium from Alginate-Encapsulated LESC

**Figure 2:**
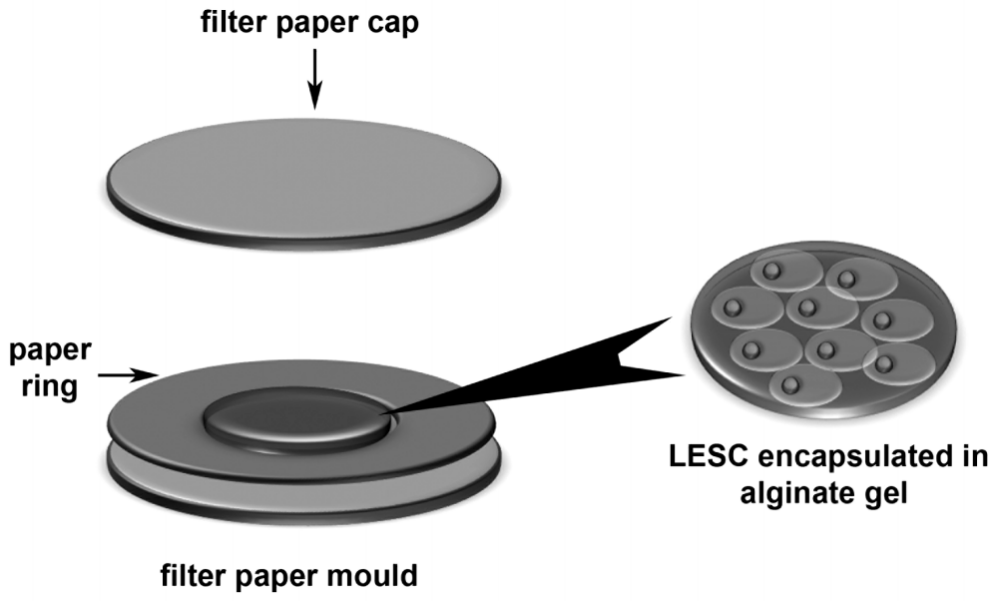
Schematic of LEC encapsulation in alginate gels. To form alginate gel discs, a paper ring with a 2 cm diameter opening is placed over a 3 cm diameter paper disc, and saturated with 102 mM CaCl_2_ before alginate solution (420 µL) was pipetted into the ring. A second 3 cm diameter paper disc saturated with 102 mM CaCl_2_ was placed over the alginate/paper assembly. Alginate solutions were exposed to 102 mM CaCl_2_ for 10 min to allow complete gelation. Dimensions of calcium alginate gel masses and discs. Calcium alginate gel discs are approximately 19 mm in length and 1.5 mm in depth.

3×10^5^ primary LESC were suspended in 1.2% w/v sodium alginate solution (420 µL) before gelling into discs as previously described [20] using 102 mM CaCl_2_. Briefly, to form gel discs, a paper ring with a 2 cm diameter opening was placed over a 3 cm diameter paper disc, and saturated with 102 mM CaCl_2_ before pipetting 420 µL alginate solution into the ring (**Figure 2**). A second 3 cm diameter paper disc saturated with 102 mM CaCl_2_ was placed over the alginate/paper assembly. Alginate solutions were exposed to 102 mM CaCl_2_ for 10 min to allow complete gelation. Calcium alginate discs with or without LESC were suspended in CnT20 medium under and cultured at 37°C, 5% CO_2_, 95% humidity for 3 days.

Conditioned medium (CM) was prepared from both alginate-encapsulated and non-encapsulated LESC ((3×10^5^) cultured on tissue culture plastic in CnT20 for 3 days). After the 3-day culture period, CM from alginate-encapsulated and non-encapsulated LESC was collected and centrifuged at 15500 g for 10 min to remove cellular debris. The supernatant was retained and the pellet discarded. CM generated from individual corneoscleral rims was not pooled and each sample represented a separate biological replicate.

#### Culture of a human corneal epithelial cell-line in LESC conditioned medium

The human corneal epithelial (HCE) cell-line [21] (referred to as corneal epithelial cells: CEC) was cultured in CM^ALG-LESC^ and CM^LESC^ on tissue culture plastic for approximately 3 days to monitor the ability of these cells to attach, form colonies and proliferate. CnT20 medium incubated with alginate gels alone (CM^ALG^), CnT20 cultured without cells (CM^CULT^), and non-cultured CnT20 (CM^NON-CULT^) were used to culture CEC as experimental controls. CEC were isolated from culture medium after the 3-day culture period, and the Trypan blue exclusion assay was performed to assess cell viability and proliferation. A 10 µL cell suspension was mixed with 10 µL Trypan blue dye solution, before counting live (unstained) and dead (stained-blue) cells using a haemocytometer.

#### One Dimensional Gel Electrophoresis

Sodium dodecyl sulphate - polyacrylamide gel electrophoresis (SDS-PAGE) was carried out in discontinuous vertical slab gels which contained a final concentration of 15% v/v acrylamide in the resolving gel and 4% (v/v) acrylamide in the stacking gel. The stacking gel contained stacking gel buffer (0.5 M Tris-base; pH 6.8), 30% (v/v) acrylamide, 10% (w/v) SDS, 0.05% (v/v) TEMED and 1.5% (w/v) AMPS. The resolving gel comprised resolving gel buffer (3 M Tris-base and 5 N HCl; pH 8.8), 30% (v/v) acrylamide, 10% (w/v) SDS, 0.08% (v/v) TEMED and 1.5% (w/v) AMPS. The amount of total protein in LESC lysates and CM samples was quantified using the modified Lowry protein assay, and samples were prepared by addition of an equal volume of Laemmli reducing sample-treatment buffer (RSTB: 4% (w/v) SDS, 10% (v/v) 2-mercaptoethanol, 20% (v/v) glycerol, 0.5 M Tris-base and 0.001% (w/v) bromophenol blue) to solutions of lysed cells and CM (1 1). RSTB-treated samples (10 µg total protein per sample) were loaded onto the stacking gel and separated in resolving gels using 100 V per gel. Gels were then either silver stained or immunoblotted.

### Silver nitrate protein staining

Proteins from LESC CM were stained with silver nitrate. Briefly, proteins immobilized in 15% v/v acrylamide gels were fixed in a solution containing 40% (v/v) methanol, 10% (v/v) acetic acid and 50% (v/v) water. Gels were then washed 3 times for 5 min each time before proteins were sensitized (5% (w/v) sodium thiosulphate, 30% (v/v/) ethanol and 68 mg/mL sodium acetate) for 30 min. The gel was then washed 3 times with deionised water for 10 min each time, and stained with 2.5% (w/v) silver nitrate for 20 min. Stained gels were washed twice with deionised water (2 min per wash, and the silver stain was developed using a solution of 2.5% (w/v) sodium carbonate and 0.04% (v/v) formaldehyde. When protein bands were visible, development of the stain was stopped using a 1.46% (w/v) EDTA-Na_22_H_2_O solution. The stained gels were washed with water imaged using a GE Healthcare gel scanner densitometer and ImageQuant software. Gels were stored in 5% (v/v) acetic acid at 4°C.

### Immunoblotting

Proteins were transferred from SDS-PAGE gels to PVDF membranes under semi-dry conditions using 200 V per gel for 2 h. Non-specific binding to membranes was blocked by incubation with 5% (w/v) bovine serum albumin (BSA) dissolved in 1× TBS-T (20 mM Tris-base, 0.14 M NaCl, 0.1% Tween®-20; pH 7.6) for 1 h at room temperature. Membranes were incubated with primary antibodies (anti-p63 1/8000 dilution), anti-sPARC, anti-galectin-1, and anti-profilin-1) diluted in 2% (w/v) BSA dissolved in 1× TBS-T at 4°C overnight. β-mercaptoethanol stripping buffer was used to remove the primary p63 antibody from membranes before an anti-14-3-3ζ antibody (1/4000 dilution) was used to detect 14-3-3ζ (loading control). Blots were washed for 45 min in 1× TBS-T before incubation with HRP-conjugated anti-rabbit (1 4000 dilution) secondary antibody for 2 h at room temperature with rotation. Proteins were detected on X-ray film using an ECL system; the film was exposed to membranes for 5–10 seconds to capture protein/ECL signals.

### Statistical Analysis

Unpaired t-tests were performed using Microsoft Excel to determine the statistical significance between CEC proliferation in conditioned medium from alginate-encapsulated and non-encapsulated LESC. Results are presented as the mean of three individual experiments with standard error of mean (S.E.M.) and *P*-value≤0.05 considered significant.

## Results and Discussion

### Corneal epithelial cell proliferation is modulated by CM from alginate-encapsulated LESC

We have previously demonstrated that calcium alginate gels are suitable for storage/transport of immobilized embryonic stem cells, mesenchymal stem cells, and LESC [20], [22], proving that these 3-D matrices are capable of retaining the progenitor phenotype of encapsulated cells.

**Figure 3:**
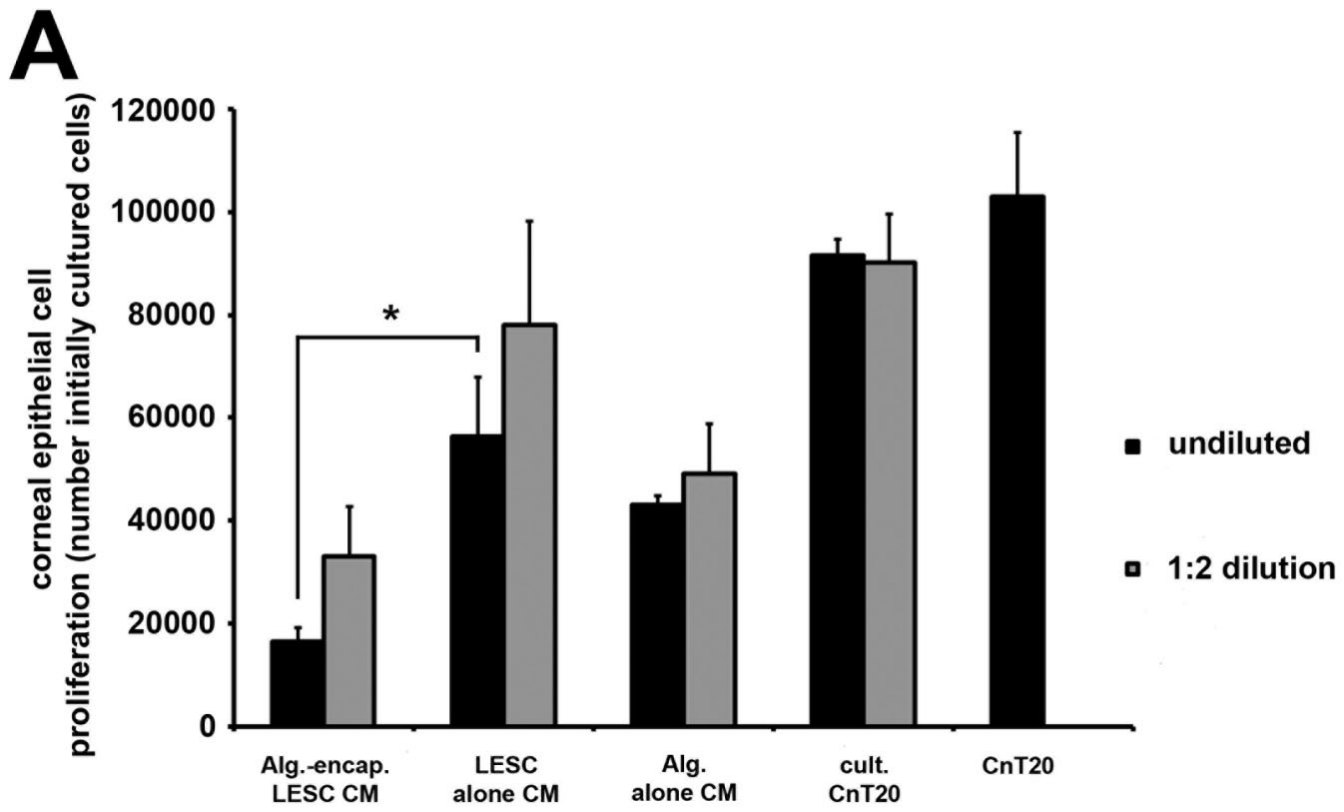
LESC conditioned medium modulates corneal epithelial cell proliferation. Data points represent the mean (n = 3±S.E.M.) number live cells extracted. * *P*≤0.05 indicate differences between culture conditions.

To determine the influence of the alginate gel matrix on the production of potential corneal wound healing factors from LESC, we examined the ability of CEC to proliferate in CM generated from alginate-encapsulated LESC (CM^ALG-LESC^) (**Figure 3**). Proliferation of CEC was attenuated significantly (*P*≤0.05) (>2 fold) in undiluted CM from CM^ALG-LESC^ compared to CEC cultured in CM from non-encapsulated LESC (CM^LESC^). The addition of CnT20 to CM^ALG-LESC^ and CM^LESC^ increased CEC proliferation, but CEC growth remained significantly lower in CM^ALG-LESC^. This indicated that despite the inclusion of growth promoting factors from CnT20 medium, inhibition of CEC growth in CM^ALG-LESC^ was not completely reversed.

The proliferation of CEC in CM^ALG^ was less pronounced than CEC proliferation in cultured CnT20 (CM^CULT^) or non-cultured CnT20 (CM^NON-CULT^). The basal media CnT20 contains factors known to promote CEC proliferation, therefore the decrease in proliferation of CEC in CM^ALG^ compared to CM^CULT^ or CM^NON-CULT^ may be due to the sequestering of factors from CnT20 medium by the alginate gel that we have shown is capable of adsorbing proteins from medium solutions (Table S1). Protein levels were significantly greater (*P*≤0.05) in alginate gels suspended in CnT20 medium with supplements (97±0.007 µg/mL) than those suspended in basal medium (75±0.004 µg/mL) (Table S1). Protein adsorption to alginate hydrogels was previously reported [23]. Therefore, diluting the CM^ALG^ with fresh CnT20 partially replenishes some of the growth factors explaining the moderate increase in proliferation. Interestingly, CEC proliferation in CM^ALG-LESC^, was significantly lower (*P*≤0.01) than that from CM^ALG^, strongly suggesting that either a factor inhibitory to proliferation was produced by encapsulated LESC or that expression and/or release of factors required for promoting CEC proliferation were prevented by the 3D gel structure.

Previously we reported that the proliferation of LESC immobilized in alginate gels is limited or prevented [20]. This was possibly due to mechanical forces exerted on encapsulated LESC, i.e. cells are physically prevented from growing (possibly due to pore sizes which were significantly smaller than the size of cells or inappropriate levels of gel stiffness); an increase in the compressive modulus of alginate gels decreased the viability of immobilized cells [20]. Studies have shown that the movement of small-molecules through alginate gels is controlled by the internal structure of these matrices [24]. Increasing alginate concentration from 1.5% to 3%, decreasing intrinsic pore size, was previously shown to hinder the movement of various solutes through this gel. Therefore, an alternative explanation may be that factors required for proliferation of CEC are released too slowly from alginate gels. The release of proteins or peptides from alginate encapsulated LESC may be dependent on their size, with larger proteins migrating at a slower rate through gels than peptides of relatively lower molecular mass.

Taken together the data presented here suggest that LESC encapsulated in an alginate gel matrix secrete factors that can attenuate the growth of a secondary cell population.

### Proteins that regulate epithelial cell proliferation are identified within the LESC secretome

To determine the possibility that factors secreted from LESC may be responsible for the observed inhibitory effect on CEC proliferation, we performed biochemical characterisation of CM^ALG-LESC^.

**Figure 4:**
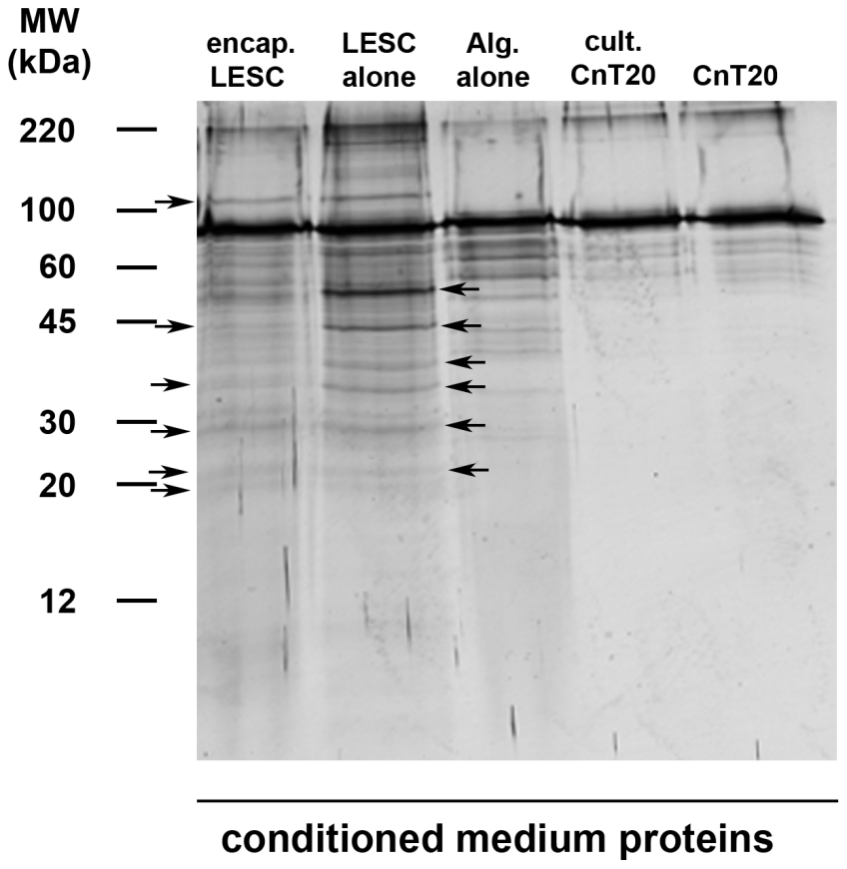
Profiles of secreted proteins from alginate-encapsulated and cultured LESC are distinct. Conditioned medium from alginate-encapsulated LESC (CM^ALG-LESC^), LESC cultured in CnT20 (CM^LESC^), alginate gels alone (CM^ALG^), cultured CnT20 (CM^CULT^), and non-cultured CnT20 (CM^NON-CULT^) were treated with Laemmli buffer containing the reducing agent β-mercaptoethanol. Treated protein mixtures were separated using 15% acrylamide gels and proteins were stained using silver nitrate. Gel represents 3 individual experiments from 3 different corneoscleral rims.

Examination of the LESC secretome by 1-D gel electrophoresis and silver staining resolved distinct protein banding patterns from CM^ALG-LESC^ and CM^LESC^ (**Figure 4**). The amount of total protein loaded onto gels were equivalent to 10 µg for each sample, therefore differences observed between samples are not biased by unequal protein loading. Proteins (indicated on **Figure 4** with arrows) ranging from approximately 35–55 kDa were expressed at lower levels in CM^ALG-LESC^ than from CM^LESC^. At lower molecular weights 30–20 kDa protein bands from both CM^ALG-LESC^ and CM^LESC^, are expressed to similar levels. Proteins included into CnT20 medium as supplementary factors (approximately 55 – 80 kDa) were expressed to greater levels in CM^ALG^, than in CM^CULT^ or CM^NON-CULT^, suggesting that medium proteins within the CnT20 basal media may be both concentrated and adsorbed (see **Figure 3**) by alginate gels.

We hypothesise that proteins ranging from approximately 35–55 kDa which varied in levels due to encapsulation within alginate gels may elicit effects on epithelial cell growth. We used previous studies investigating the secretome of epithelial cells from non-pigmented ciliary [25] and mammary tissue [26] as mining references for proteins of potential relevance to corneal wound healing. The secretome of non-pigmented ciliary epithelium was of particular interest as this group of proteins was studied as candidate factors for promoting differentiation and growth of retinal ganglion cells [25]. Based on this study we focused on identifying thrombospondin 1, galectin-1, secreted protein, acidic and rich in cysteine (sPARC), cofilin-1, profilin-1, and myotrophin in CM^ALG-LESC^, as these proteins play important roles in cell proliferation and/or differentiation.

**Figure 5:**
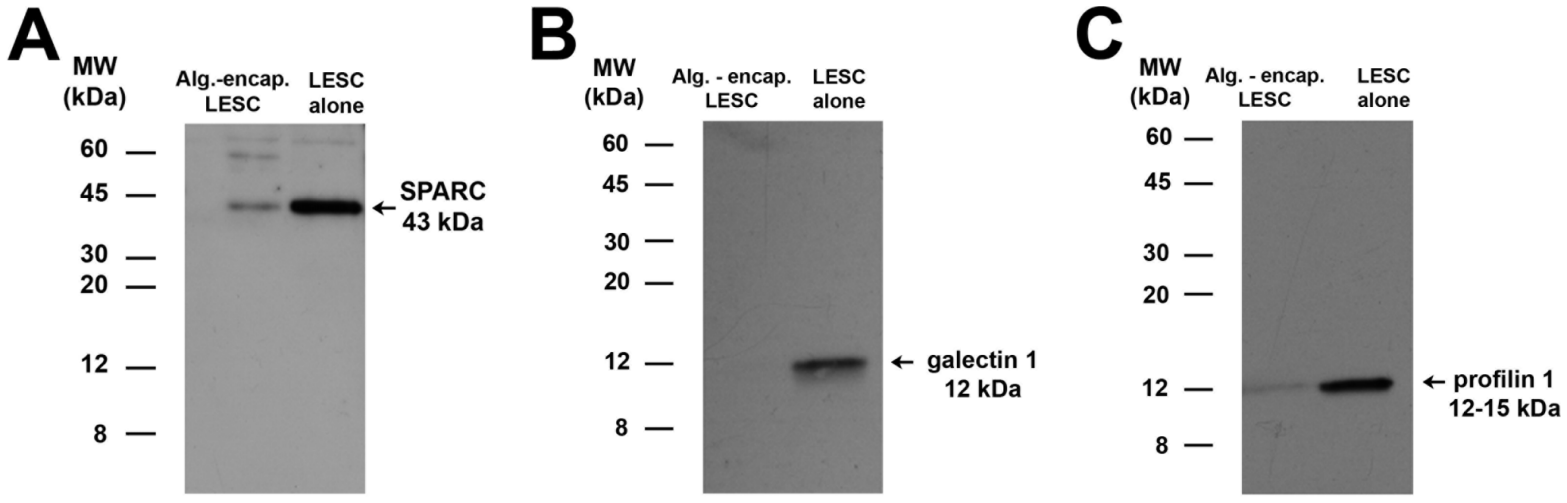
Proteins known to modulate cell proliferation are identified within the LESC secretome. SPARC (A), galectin 1 (B), and profilin 1 (C) were detected in conditioned medium from alginate-encapsulated LESC (CM^ALG-LESC^) and LESC (CM^LESC^) cultured in CnT20 for 3 days under cell culture conditions by immunoblotting. Blots represent 3 individual experiments from 3 different corneoscleral rims.

We identified by Western blotting, sPARC, profilin-1, and galectin-1 in the CM^ALG-LESC^ and CM^LESC^ (**Figure 5**). Samples were equally loaded: 10 µg total protein from CM^LESC-ALG^ and CM^LESC^ were loaded separately onto gels. sPARC (**Figure 5A**), a 43 kDa protein (see 43 kDa band indicated on **Figure 4**), that was identified in CM^LESC-ALG^ and CM^LESC^ was particularly interesting due to well-characterised functions as a growth factor modulator and cell cycle inhibitor [27]. sPARC may therefore, be responsible, at least in part, for the marked inhibition of CEC proliferation in CM^LESC-ALG^. However, the greater proliferation of CEC in CM^LESC^ than CM^ALG-LESC^ may be due to galectin-1 (**Figure 5B**) that induces cell proliferation [28]. This protein was highly expressed in CM^LESC^ but was not expressed in CM^ALG-LESC^.

**Figure 6:**
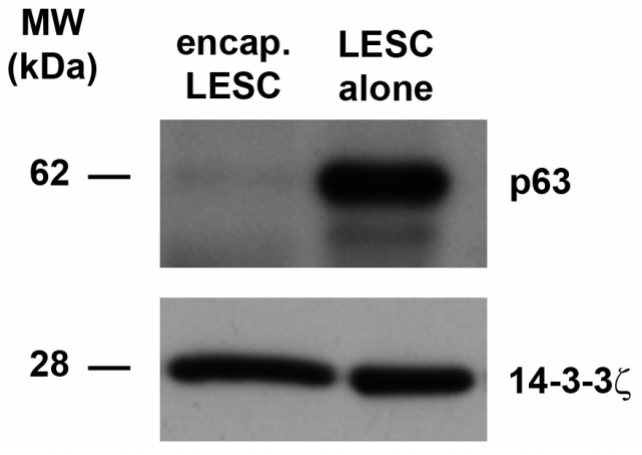
Cultured LESC retain a progenitor phenotype. P63 was detected in freshly isolated LESC (control: C), alginate-encapsulated LESC (Alg) and LESC cultured (Cult) in CnT20 for 3 days under cell culture conditions by immunoblotting. 14-3-3ζ was used as a loading control. Blots represent 3 individual experiments from 3 different corneoscleral rims.

Encapsulated LESC expressed the progenitor marker, p63, (**Figure 6**) to lower levels than cultured LESC indicating that these cells were potentially undergoing differentiation within alginate gels. Profilin-1 (**Figure 5C**) that was shown to be secreted by CM^ALG-LESC^ was recently demonstrated as an essential element for the differentiation of human epithelial cells [29]. Alginate-encapsulated LESC may be at various stages of differentiation dependent on surrounding levels of profilin-1. Although the secreted lectin, galectin-1 (**Figure 5B**) was not identified in CM^ALG-LESC^, this protein may also drive differentiation of corneal epithelial cells.

sPARC may regulate the differentiation of epithelial cells as well as the inhibition of these cells during corneal wound healing. Disruption of the sPARC locus in mice with sPARC-null lens resulted in altered lens development manifested by changes in epithelial cell differentiation [30]. sPARC was reported to be secreted from corneal epithelial cells, but this protein was only shown to regulate the contraction of keratocytes embedded in collagen gels [31]. Similar to our study, this study confirmed using Western blot analysis that sPARC was contained in epithelial cell conditioned medium. The authors reported that collagen gel contraction by keratocytes with increased contractility due to addition of purified sPARC was enhanced in a dose-dependent manner. Furthermore, sPARC was identified in the basal layer of the migrating epithelium and activated keratocytes adjacent to the wound 3 days and 1 week after perforating injury in rabbit corneas.

sPARC-mediated mechanisms for inhibition of CEC proliferation may involve inhibition of IGF-dependent signalling [10] and stimulation of the TGF-β signalling pathway [32] both at the receptor level and within cells. sPARC has been reported to inhibit epithelial cell proliferation by stimulating the TGF-β signalling system [32], and this effect is corrobated by the Mishima *et al.* (1998) [31] study that describes a direct effect of sPARC on fibroblasts, i.e. scar contraction. As TGF-β transforms stromal keratocytes into myofibroblasts that are the primary mediators of scar formation [11], it is thought that reducing levels of sPARC in the cornea could limit or eliminate scar formation and enhance closure of corneal epithelial wounds.

Reports showing sPARC inhibition of IGF signalling demonstrated that sPARC-null mesangial cells producing increased amounts of IGF-1 and -2, as well as IGF-1 receptor (IGF-1R) were attenuated by the addition of recombinant sPARC [10]. The secreted protein inhibited sPARC-null cells by blocking IGF-1-stimulated mitogen activated protein kinase (MAPK) activation and DNA synthesis. An accelerated rate of basal and IGF-1-stimulated proliferation in mesangial cells derived from sPARC-null animals was shown to be partially due to significantly diminished levels of cyclin D1 and the cyclin-dependent kinase (CDK) inhibitors p21 and p27. As IGF signalling in the cornea stimulates epithelial cell proliferation [33], sPARC is likely to play dual complementary roles in corneal wound healing that culminate in inhibition of epithelium turnover and reinforcement of scar tissue.

**Figure 7:**
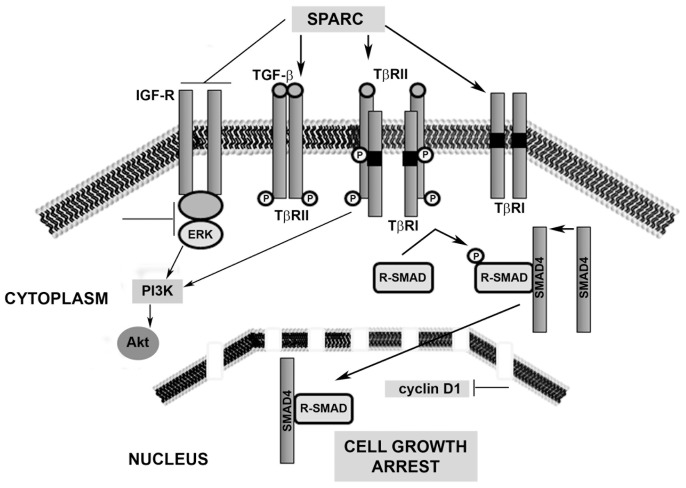
A working model of LEC secretome mechanisms for the modulation of epithelial cell proliferation. SPARC may inhibit corneal epithelial cell proliferation by targeting IGF, TGF-β and Smad2/3 signalling that is established in corneal wound healing. In the corneal epithelium, sPARC may inhibit the IGF receptor and activate the TGF-β. Inhibition of epithelium turnover may occur through sPARC-mediated attenuation of extracellular regulated kinase (ERK), phosphoinositide-3-kinase (PI3K) and Akt in the cytoplasm leading to reduction in the activity of the cell cycle regulator, cyclin D1 in the nucleus.

A working model (**Figure 7**) of novel wound healing processes in the cornea could be developed, demonstrating that sPARC affects intracellular events downstream of TGF-β and IGF receptors by directly targeting these membrane proteins and subsequently downstream signalling proteins within cells including ERK, PI3K and Akt as well as cell cycle regulator, cyclin D1. Together, these mechanistic events may be the drivers that ultimately lead to growth arrest of CEC.

Collectively, these data suggest that the secretome of alginate-encapsulated LESC may be applied to corneal dysfunctions requiring control of the growth of epithelium.

## Conclusions

We have demonstrated that LESC secrete proteins that exert powerful effects on corneal epithelial growth. Our alginate gel matrix allows the secretion of inhibitory proteins from encapsulated LESC that may regulate epithelialisation of the cornea. Corneal wound healing is complex and stringently regulated, but we describe findings that indicate the potential for delivering molecules produced from LESC to modulate repair of the injured cornea using bioartificial alginate hydrogel systems. These gels are very accessible for this application, as selection of the type of alginate and coating agent, the pore size, and gel degradation rate, would ultimately control release kinetics of cell-derived soluble factors. Therefore these matrices as bioartificial niche micro-environments can be applied to clinical distribution of endogenous LESC-derived proteins in a manner that mimics their physiological rate of release. The present study may lead to the discovery of novel facets of corneal wound healing dynamics that may be dependent on secreted proteins derived from both terminally-differentiated and progenitor corneal epithelial cells.

## Supporting Information

Table S1 **Alginate gels adsorb proteins from CnT20 medium.** Samples represent the mean (n = 3±S.E.M.) amount of protein from supplemented and basal CnT20 medium and alginate gels suspended in those media.
(DOCX)
