# Supplementary material for: The Secretome of Alginate-Encapsulated Limbal Epithelial Stem Cells Modulates Corneal Epithelial Cell Proliferation
Source: PLoS One. 2013 Jul 24;8(7):e70860. doi: 10.1371/journal.pone.0070860 (PMC3722209; doi:10.1371/journal.pone.0070860)
Supplement: Table S1 — Alginate gels adsorb proteins from CnT20 medium. Samples represent the mean (n = 3±S.E.M.) amount of protein from supplemented and basal CnT20 medium and alginate gels suspended in those media. (DOCX) [file pone.0070860.s001.docx]

| **sample** | **protein concentration**  **(µg/mL) (± S.E.M.)** |
| --- | --- |
| supplemented CnT20 medium | 353 ± 0.006 |
| basal CnT20 medium | 251 ± 0.004 |
| alginate gel from supplemented CnT20 | 97 ± 0.007 |
| alginate gel from basal CnT20 | 75 ± 0.004 |
